# A randomized study on migration of the Spectron EF and the Charnley flanged 40 cemented femoral components using radiostereometric analysis at 2 years

**DOI:** 10.3109/17453674.2011.618914

**Published:** 2011-11-24

**Authors:** Thomas Kadar, Geir Hallan, Arild Aamodt, Kari Indrekvam, Mona Badawy, Leif Ivar Havelin, Terje Stokke, Kristin Haugan, Birgitte Espehaug, Ove Furnes

**Affiliations:** ^1^Department of Orthopaedic Surgery, Haukeland University Hospital, Bergen; ^2^Hagevik Hospital, Haukeland University Hospital, Hagavik; ^3^Department of Orthopaedic Surgery, Trondheim University Hospital, Trondheim; ^4^Department of Neuroscience, Norwegian University of Science and Technology, Trondheim; ^5^Department of Radiology, Haukeland University Hospital, Bergen; ^6^Department of Surgical Science, University of Bergen, Bergen, Norway

## Abstract

**Background and purpose:**

We performed a randomized study to determine the migration patterns of the Spectron EF femoral stem and to compare them with those of the Charnley stem, which is regarded by many as the gold standard for comparison of implants due to its extensive documentation.

**Patients and methods:**

150 patients with a mean age of 70 years were randomized, single-blinded, to receive either a cemented Charnley flanged 40 monoblock, stainless steel, vaquasheen surface femoral stem with a 22.2-mm head (n = 30) or a cemented Spectron EF modular, matte, straight, collared, cobalt-chrome femoral stem with a 28-mm femoral head and a roughened proximal third of the stem (n = 120). The patients were followed with repeated radiostereometric analysis for 2 years to assess migration.

**Results:**

At 2 years, stem retroversion was 2.3° and 0.7° (p < 0.001) and posterior translation was 0.44 mm and 0.17 mm (p = 0.002) for the Charnley group (n = 26) and the Spectron EF group (n = 74), respectively. Subsidence was 0.26 mm for the Charnley and 0.20 mm for the Spectron EF (p = 0.5).

**Interpretation:**

The Spectron EF femoral stem was more stable than the Charnley flanged 40 stem in our study when evaluated at 2 years. In a report from the Norwegian arthroplasty register, the Spectron EF stem had a higher revision rate due to aseptic loosening beyond 5 years than the Charnley. Initial stability is not invariably related to good long-term results. Our results emphasize the importance of prospective long-term follow-up of prosthetic implants in clinical trials and national registries and a stepwise introduction of implants.

Femoral stem loosening in cemented total hip arthroplasty (THA) is a multifactorial process with different mechanisms (Gruen et al. 1979, Barrack 2000). Factors such as the material, design, and surface finish are of fundamental importance for the long-term performance of cemented femoral hip implants (Scheerlinck and Casteleyn 2006). The longevity of cemented femoral stems has been related to the quality, stability, and endurance of the bonding between stem and cement (Chang et al. 1998, Scheerlinck and Casteleyn 2006). Different femoral stem designs have been developed to obtain increased fixation at this interface, since debonding between the cement and stem is an important mechanism in the initiation of loosening (Jasty et al. 1991).

The satin-finish Spectron femoral stem has been one of the best performing stems in the Swedish National Arthroplasty Register (Malchau et al. 2002). A modified, proximally roughened version of the Spectron stem, the Spectron EF (Smith and Nephew, Memphis, TN), was introduced in 1989 to enhance stem-cement bonding.

The use of this implant gained increasing popularity, and in 2007 the Spectron EF stem used with the Reflection All-Poly acetabular cup (Smith and Nephew) was the most commonly used primary total hip prosthesis in Norway (Espehaug et al. 2009).

The degree of migration during the first years after surgery has been shown to correlate with the long-term performance of joint prostheses (Kärrholm et al. 1994, Kobayashi et al. 1997). Radiostereometric analysis (RSA) allows the accurate measurement of implant movement and has been extensively used for measurement of the in vivo migration of implants (Kärrholm et al. 1997).

An earlier prospective randomized study reported an increased revision rate of the Charnley stem compared to the satin-finished Spectron stem (Garellick et al. 1999). In the present randomized, controlled clinical trial we wanted to evaluate the early migration of the successor to this stem, the Spectron EF stem and to compare it to that of the Charnley stem using RSA. The null hypothesis was that the migration of the Spectron EF stem was equal to that of the Charnley prosthesis (DePuy International Ltd., Leeds, UK), which has the longest follow-up and the largest volume of documentation of implants used for primary total hip arthroplasty (Aamodt et al. 2004).

## Patients and methods

The trial was registered with ClinicalTrials.gov (NCT 00698672). It was conducted according to the Helsinki Declaration and was approved by the Regional Ethical Committee.

150 patients aged 59–80 years with primary or secondary osteoarthritis of the hip were included in the study between November 2004 and June 2007 (Table 1). Each patient provided informed consent. In bilaterally operated patients, only one hip was included. Exclusion criteria were body mass index over 35, uncompensated cardiopulmonary disease, malignant disease, dementia, dysplasia with the need for bone grafts, Paget's disease, Charcot's disease, rheumatoid arthritis, or other serious systemic diseases.

**Table 1.** Baseline characteristics of included patients according to allocated treatment

	Charnley flanged 40	Spectron EF
	n = 30	n = 120
Male/female (n)	10/20	35/85
Side (right/left) (n)	16/14	59/61
Mean age	70	69
(range)	(60–80)	(59–80)
Mean weight (kg)	76	76
(range)	(53–110)	(46–106)
Charnley class		
(A/B/C) (n)	17/11/2	64/52/4
Primary/secondary arthrosis (n)	28/2	101/19 **^a^**
Mean HHS preoperatively	45	44

**^a^** 2 patients were diagnosed as having osteonecrosis of the femoral head.

**Figure 1.**
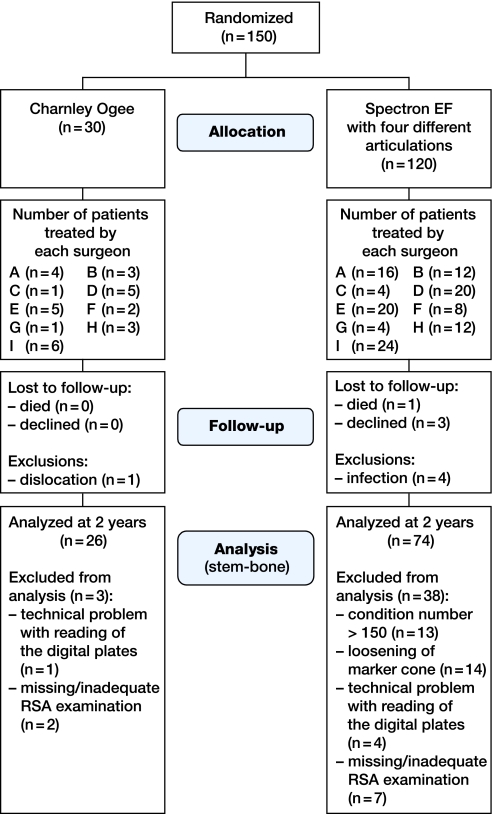
Flow chart of the patients.

This study was undertaken as a part of a study investigating wear of 5 different articulations (Kadar et al. 2011). Patients were randomized into 5 groups. One group received the Charnley femoral stem and a Charnley Ogee cup, and the other groups received the Spectron EF femoral stem with 4 different articulations (see below). Thus, 30 hips were included in the Charnley group and 120 hips in the Spectron EF group. Block randomization was used to ensure that each surgeon operated an equal number of hips in each of the 5 groups. After randomization, there were no crossovers. 8 consultant orthopedic surgeons (A, B, C, D, E, F, G, and H) and 1 resident surgeon (I) either performed or supervised the operations (Figure 1). Randomization was by sealed envelopes revealing study group. An equal number of envelopes from each study group were placed inside a larger envelope. The latter envelope was assigned to one surgeon. After inclusion of a patient, the surgeon drew out one of the smaller envelopes from the larger one. After using all the smaller envelopes, the surgeon had included an equal number of patients in each group.

The patients were blinded. The surgeons were not because of the different designs and operative techniques of the implants. The follow-up period was 24 months, with scheduled follow-up visits at 3, 6, 12, and 24 months.

### Intervention

**Figure 2.**
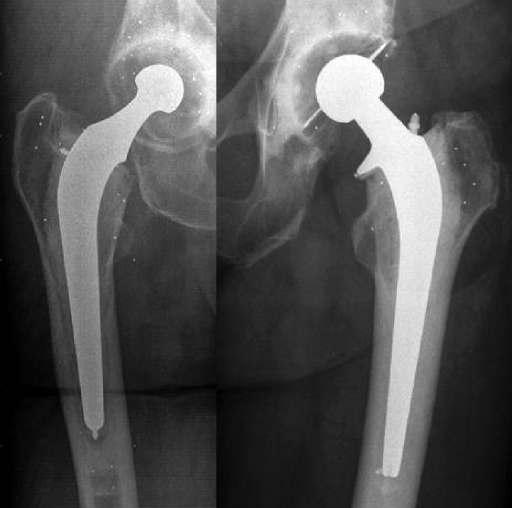
The Charnley flanged 40 (left) and the Spectron EF (right), with tantalum markers.

The patients received one of the following cemented THAs (Figure 2):

- Charnley flanged 40 monoblock, oval, stainless steel, vaquasheen surface (Ra 0.8 µm) femoral stem with a 22.2-mm head. It articulated with a cemented Charnley Ogee UHMWPE acetabular cup, which was γ-sterilized in nitrogen (DePuy International Ltd.). (n = 30)
- Spectron EF modular, square, matte distal surface (Ra 0.7 µm), collared, cobalt-chrome femoral stem with a grit blasted roughened surface (Ra 2.8 µm) on the proximal third of the stem. It was used with either an Oxinium or a cobalt-chrome 28-mm femoral head and articulated with either a cemented Reflection All-Poly or a Reflection All-Poly XLPE acetabular cup (Smith and Nephew) (n = 120).

Both stems rely on the composite-beam fixation model, in which the stem needs to be firmly bound to the cement (Scheerlinck and Casteleyn 2006).

Only patients suitable to receive both the Spectron EF stem size 2–5 with a standard offset and the Charnley flanged 40 stem were included in the study. This was determined using templates on the preoperative radiograph before inclusion.

Both femoral components were supplied with tantalum markers by the manufacturer. The Charnley flanged 40 was supplied with 2 tantalum markers. The center of the femoral head was used as a third point for the calculations of movements of the stem. The Spectron EF stem was supplied with 3 tantalum markers. One of the tantalum markers was attached to a cone, which had to be impacted into a hole in the neck of the stem after implantation. 4 tantalum markers (1 mm in diameter) were inserted into the greater trochanter, 1 into the calcar medially and 1 into the lesser trochanter. 6 tantalum markers (0.8 mm in diameter) were inserted into the cement.

Third-generation cementing technique (entering the femoral canal via fossa piriformis, high-pressure lavage, femoral cement restrictor, retrograde cement injection with a cement gun, and cement pressurization) was used in all patients. Initially, the components were inserted with Palacos R with gentamicin cement (Schering-Plough; Labo N.V., Heist-Op-Den-Berg, Belgium). After August 2005, the components were inserted with Palacos R+G cement (Heraeus Kulzer GmbH, Hanau, Germany). The bone cement was stored at 8°C in a refrigerator and taken out immediately before use. Mixing was done using the Optivac system (Biomet, Warsaw, IN). Insertion of the femoral stem was performed 5 min after mixing of the cement. Before insertion, the cement was pressurized for 1 min. Further details of the surgical procedure have been described previously (Kadar et al. 2011).

### Objectives and outcomes

Clinical outcome was evaluated with the Harris hip score (HHS) preoperatively and at 3, 12, and 24 months. The preoperative scoring was done by the operating surgeon whereas the subsequent scoring was mainly done by the first author (TK). Complications and reoperations were noted.

The median time after surgery of the index RSA examination was 11 (9–15) days. RSA examinations were repeated at 3, 6, 12, and 24 months after surgery. All RSA examinations were performed by one radiographer (TS). We used a uniplanar technique with the calibration cage positioned under the examination table (cage 43; RSA Biomedical, Umeå, Sweden). The patient was supine during examination. One gantry-mounted X-ray tube and one portable X-ray tube were used to obtain simultaneous exposures. For radiographic imaging, we used high-definition digital plates (Agfa CR MD 4.0) and for plate reading we used the ADC compact digitizer (Agfa).

The investigators involved in the RSA measurements (KH and AA) were not blinded. RSA measurements were only done if 3 or more markers in each segment could be identified on repeated examinations. Migration and rotation were calculated along and around the horizontal (x-), longitudinal (y-), and sagittal (z-) axes on the basis of signed values, and were computed by using UmRSA Digital Measure version 5.0 software (RSA Biomedical). Left hips were converted to right hips. To ensure proper stability and distribution of the tantalum markers, the upper limit for the mean error of body fitting was set at 0.35 and that for the condition number at 150 (Valstar et al. 2005).

We investigated the radiographs and HHS of patients with subsidence exceeding 1 mm and/or internal rotation of more than 3° at 2 years separately. According to an earlier study, subsidence of 1–2 mm during the first 2 years postoperatively indicates an increased risk of revision (Kärrholm et al. 1994). To our knowledge, no suggestions for limit of tolerable retroversion have been presented. However, a discretional limit of 3° was choosen for internal rotation, based on a previous study (Hallan et al. 2006).

### Statistics

For determination of the precision of the RSA measurements, the difference between double measurements on 50 patients was computed. Secondly, we calculated the standard deviation (SD) of the differences with respect to zero (Ranstam et al. 2000).

The precision (Table 2) was then calculated using the formula:


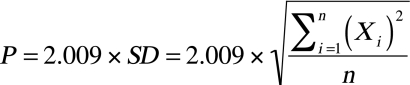


**Table 2.** The precision of RSA obtained by 50 double examinations at 1 year

	Migration of stem	Rotation of stem
	(mm)	(degrees)
x-axis	0.09	0.36
y-axis	0.13	0.76
z-axis	0.23	0.13

where P = precision, x = the difference between double examinations, and 2.009 represents the critical value at a two-sided 95% t-distribution for a sample size of 50.

Small numbers of subjects, 15–25 patients, can be used in RSA studies as a consequence of the high precision of the measurement technique (Valstar et al. 2005). At the start of this study, the patients were simultaneously enrolled in a study of 5 different articulations. We anticipated some exclusions and missing or imperfect RSA examinations. 30 patients were therefore included in each group, resulting in 30 patients in the Charnley group and 120 patients, with 4 different articulations, in the Spectron EF group.

The effects of the different prostheses and the time on the RSA migration data were investigated in a linear mixed-effect model, which took into account any correlation in outcome measures introduced by the repeated-measures design. Assumptions for these analyses were assessed based on residual plots and normal probability plots of the residuals and of the estimated random effects (Pinheiro and Bates 2001). One-way analysis of variance was used to determine whether the mean migration at 2 years differed significantly among the 4 different articulations in the Spectron EF group.

Differences were regarded as being statistically significant if the p-value (two-sided) was less than 0.05. Statistical evaluation was performed using SPSS software version 17.0.

## Results

### Follow-up

Of 150 patients recruited, 4 were lost to follow-up and 5 were excluded for dislocation or infection at 24 months. 41 (27%) of the expected RSA examinations between bone and prosthesis at 2 years were excluded due to a high condition number, loosening of the marker cone on the Spectron EF prosthesis, a transient technical problem with the reading of the digital plates, deficiency of a sufficent number of visible markers at the RSA examinations, or missing RSA examinations (Figure 1).

We performed measurements of the cement mantle with the Spectron EF stem. Because of the relatively thin cement mantle and the rectangular and voluminous proximal stem design, the visibility of the cement markers was difficult and only 26 hips could be measured at 2 years.

### Clinical results

In the Charnley group, the mean HHS (SD) increased from 45 (13) points preoperatively to 91 (11) points at 2 years, similarly to that in the Spectron EF group: 44 (15) and 91 (10).

### Radiostereometric analysis

The femoral components in both groups subsided, migrated posteriorly, and rotated into retroversion. The mean subsidence (migration along the y-axis) (SD) at two years was –0.26 mm (0.42) for the Charnley group and –0.20 mm (0.44) for the Spectron EF group (p = 0.5).

**Figure 3.**
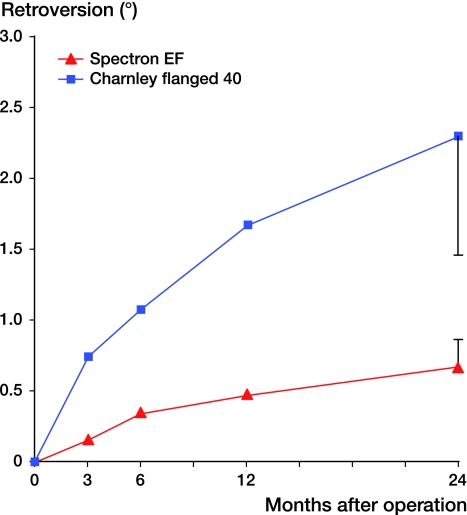
Observed mean values of retroversion (rotation around the y-axis) of the femoral components (with 95% confidence interval of mean).

**Figure 4.**
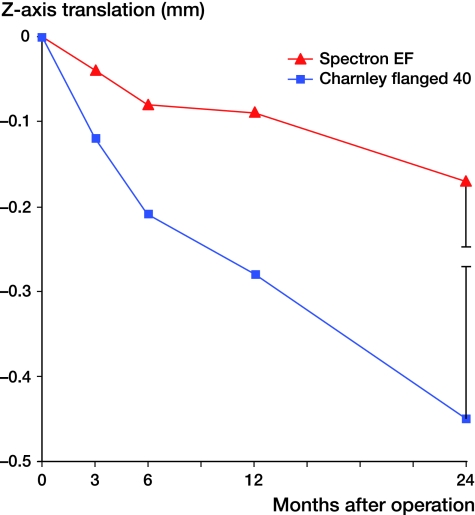
Observed mean values of posterior translation (negative translation along the z-axis) of the femoral components (with 95% confidence interval of mean).

Mean internal rotation (retroversion) was 2.3° (2.2) and 0.7° (0.9) (p < 0.001) (Figure 3), and posterior translation (negative translation along the z-axis) was –0.45 mm (0.48) and –0.17 (0.35) mm (p = 0.002) (Figure 4) for the Charnley and Spectron EF groups, respectively. Migrations in the other directions were minor (Tables 3 and 4).

**Table 3.** Time-dependent differences in RSA-measured micromovement between stem and bone, by stem prosthesis, estimated in a mixed linear regression model

	Charnley			Spectron EF			Test for
	Mean **^a^**	Beta **^b^**	p-value	Mean **^a^**	Beta **^b^**	p-value	difference **^c^**
Rotation x (°) (SD)							
3 months	0.05 (0.34)	0.09	0.1	–0.00 (0.25)	0.08	0.02	
6 months	0.11 (0.40)	0.15	0.01	–0.07 (0.34)	0.00	0.9	
12 months	0.02 (0.48)	0.06	0.1	–0.08 (0.46)	0.00	0.9	
24 months	–0.04 (0.58)	Ref.		–0.08 (0.4)	Ref.		0.6
Rotation y (°) (SD)							
3 months	0.74 (0.53)	–1.6	< 0.001	0.16 (0.41)	–0.52	< 0.001	
6 months	1.1 (1.1)	–1.3	< 0.001	0.35 (0.51)	–0.34	0.001	
12 months	1.7 (1.6)	–0.63	< 0.001	0.48 (0.93)	–0.17	0.1	
24 months	2.3 (2.2)	Ref.		0.67 (0.89)	Ref.		< 0.001
Rotation z (°) (SD)							
3 months	–0.09 (0.11)	0.07	0.02	–0.03 (0.14)	–0.01	0.8	
6 months	–0.08 (0.18)	0.06	0.03	–0.04 (0.19)	–0.01	0.5	
12 months	–0.14 (0.19)	0.01	0.7	–0.03 (0.19)	–0.00	1.0	
24 months	–0.14 (0.24)	Ref.		–0.03 (0.19)	Ref.		0.01

**^a^** Observed mean micromovement (SD).

**^b^** Estimated regression coefficient for differences in micromovement measured relative to values at 2-year follow-up.

**^c^** p-value for test of group differences in mean values.

**Table 4.** Time-dependent differences in RSA-measured micromovement between stem and bone, by stem prosthesis, estimated in a mixed linear regression model

	Charnley			Spectron EF			Test for
	Mean **^a^**	Beta **^b^**	p-value	Mean **^a^**	Beta **^b^**	p-value	difference **^c^**
Translation x (mm) (SD)							
3 months	0.03 (0.06)	–0.01	0.6	–0.02 (0.07)	–0.00	0.9	
6 months	0.02 (0.09)	–0.01	0.4	–0.02 (0.08)	–0.00	0.6	
12 months	0.01 (0.10)	–0.02	0.1	–0.01 (0.09)	0.00	0.6	
24 months	0.03 (0.12)	Ref.		–0.02 (0.11)	Ref.		0.03
Translation y (mm) (SD)							
3 months	–0.07 (0.07)	0.19	0.01	–0.06 (0.12)	0.13	0.001	
6 months	–0.11 (0.11)	0.15	0.01	–0.10 (0.18)	0.10	0.004	
12 months	–0.18 (0.24)	0.08	0.03	–0.15 (0.28)	0.04	0.04	
24 months	–0.26 (0.41)	Ref.		–0.20 (0.44)	Ref.		0.52
Translation z (mm) (SD)							
3 months	–0.12 (0.11)	0.33	< 0.001	–0.04 (0.14)	0.13	0.001	
6 months	–0.21 (0.20)	0.24	< 0.001	–0.08 (0.18)	0.09	0.003	
12 months	–0.28 (0.32)	0.17	< 0.001	–0.09 (0.28)	0.07	0.001	
24 months	–0.45 (0.48)	Ref.		–0.17 (0.35)	Ref.		0.002

**^a^** ^–^ **^c^** See Table 3.

Between 12 and 24 months, the Charnley flanged 40 rotated 0.6° into retroversion (p < 0.001). There was no significant retroversion of the Spectron EF between 12 and 24 months (p = 0.1). Both components continued to migrate posteriorly and to subside between 12 and 24 months (p < 0.05) (Tables 3 and 4).

One-way analysis of variance showed that the migration of the Spectron EF stem was not affected by the use of different articulations (p = 0.1–0.9).

Micromovements of the cement mantle relative to the surrounding bone were of minor magnitude with the Spectron EF stem at 2 years (n = 26). The mean distal migration (SD) of the cement mantle was –0.02 mm (0.06). The mean internal rotation was 0.07° (1.0) and the mean posterior translation was –0.07 mm (0.3).

We found no influence of cement brand on stem migration.

8 stems rotated more than 3° (3.2–9.5). 3 of these also subsided more than 1 mm (range 1.9–3.4). All of these patients had HHS at 2 years of more than 80, except for 1 Spectron EF prosthesis with a low HHS of 63. Plain radiographs of this patient at 2 years showed progressive radiolucent lines in Gruen zones 1, 3, 4, 5, and 7. The patient had no pain in the thigh or in the groin, but complained of trochanteric pain and gluteal muscle insufficiency. None of the other patients analyzed separately had radiographic signs of loosening. Data on migration from patients with excessive migration were not excluded from the overall analysis.

## Discussion

The Charnley flanged 40 femoral stem was less stable than the Spectron EF stem when evaluated at 2 years. Our results suggest that the cement mantle was stable in the Spectron EF group. Another study on the Spectron EF stem found that migration was mainly translation and rotation of the stem relative to the cement mantle (Digas et al. 2005). A previous trial with the Charnley stem at our institution found a stable cement mantle (Hallan et al. 2006). We therefore attribute the magnitude of migration in the present study to translation and rotation of the stem relative to the cement mantle.

In this study, both femoral stems had a magnitude of subsidence that was within the limits of what is considered to be safe with respect to long-term performance (Kärrholm et al. 1994, Kobayashi et al. 1997).

Our findings of higher stability of the proximally roughened and rectangular cross-sectioned Spectron EF femoral stem, as compared to the Charnley flanged 40 stem, is in accordance with earlier reports on surface roughness and stem geometry (Scheerlinck and Casteleyn 2006, Verdonschot and Huiskes 1998). A low early revision rate has been reported for the Spectron EF stem (Thien and Kärrholm 2010). Given the supposed predictive nature of RSA studies, one might also anticipate that the Spectron EF would have superior long-term results. However, a report from the Norwegian Arthroplasty Register has described inferior results for the Spectron EF stem compared to the Charnley (Espehaug et al. 2009). Beyond 5 years of follow-up, the Spectron EF had a higher revision rate due to aseptic loosening than the Charnley stem. Our results at 2 years are therefore not consistent with the medium-term results presented in the register study.

The findings in the register might be explained by the differences in design and surface of the prostheses, as these factors could influence the mechanism of failure in the long term. The durability of cemented THA could be expected to be increased for stems that firmly bond to their cement mantles, and thus have small degrees of migration within the cement mantle. However, the bond between the stem and the cement mantle can normally not be maintained for the complete lifetime of the implant (Jasty et al. 1991). Thus, not just the bonding strength, but also the effects of debonding of different implants should be considered. It has been reported that the interface might be stronger with a rough surface, which may postpone debonding, but when they debond rough stems may produce more cement debris than polished ones (Verdonschot and Huiskes 1998). Debonding also reduces the cement-implant conformity and can accelerate micromotions at the cement-bone interface. The results from an RSA study have suggested that many Spectron EF stems debond from the cement mantle (Digas et al. 2006). 2 reports have shown that debonded Spectron EF femoral stems cause fretting between the rough metallic surface and the cement, which results in abrasion and release of metal and cement debris (Gonzalez et al. 2006, Grose et al. 2006). To our knowledge, these observations have not been reported with the satin finish-surfaced older version of the Spectron stem.

The failure mechanism of debonding, fretting, and loosening takes time and might explain the inconsistency of our short-term follow-up study and the longer-term results of the register study concerning the Spectron EF femoral stem. However, it is important to acknowledge that the results for the Spectron EF stem in the register study may have been adversely affected by the relatively poor results of the Reflection cup that was used together with the stem in the majority of cases. Some stem revisions were probably done to facilitate the removal of a loose or worn cup, and some stems were probably found to be loose at surgery—although not radiographically or clinically suspected to be loose preoperatively. Thus, the poor cup results may skew the results of the stem.

Subsidence and retroversion of the Spectron EF stem in this study might be interpreted as being slightly higher than in other studies of the same stem (Digas et al. 2005, Olofsson et al. 2006). We found a slightly higher degree of subsidence, retroversion, and posterior translation of the Charnley flanged 40 femoral component than was found in a previous study from our institution (Hallan et al. 2006). Overall, we do not consider our results to differ much from those presented in previous studies.

Patient-related confounding factors were minimized by the study design. RSA is a validated method to measure migration in THA, and we followed the proposed guidelines for standardization of the method (Valstar et al. 2005). The RSA examinations are technically difficult to perform and we planned our study to allow for exclusions. We encountered a problem with loosening of the marker cone on the Spectron EF neck due to insufficent fixation. This problem was identified early in the study, however, and measures were taken to avoid this problem.

### Conclusion

We found significant differences in migration patterns between the Spectron EF and Charnley femoral stems. The Charnley stem migrated more into retroversion and posterior translation.

In vivo migration patterns at 2 years should, however, be considered in combination with the surface finish and design of the implant, as these factors influence the modes of failure.

Although no conclusions can be drawn regarding the role of surface finish and stem design in the present study, these factors might explain the promising early RSA results and the unfavorable medium-term results from the Norwegian arthroplasty register regarding use of the Spectron EF stem.

Initial stability is not invariably related to good long-term results. Our results emphasize the importance of prospective long-term follow-up of prosthetic implants in clinical trials and national registries, and stepwise introduction of implants.
